# The Toxic Effect and Mechanism of TMZ Combined with siHOXB9 on Glioblastoma Cells

**DOI:** 10.3390/ijms27010079

**Published:** 2025-12-21

**Authors:** Xiaoyu Liu, Yunfei Liu, Wenxuan Li, Qianwen Wang, Ziyu Huang, Xiyu Cheng, Qiong Yan, Honggang Hu

**Affiliations:** Department of Physical Science and Engineering, Beijing Jiaotong University, Beijing 100091, China; 20121609@bjtu.edu.cn (X.L.); 23121761@bjtu.edu.cn (Y.L.); 21121601@bjtu.edu.cn (W.L.); 23121764@bjtu.edu.cn (Q.W.); 25121544@bjtu.edu.cn (Z.H.); xycheng@bjtu.edu.cn (X.C.)

**Keywords:** temozolomide, solid lipid nanoparticle, glioblastoma, U251 cell, proteomics

## Abstract

Glioblastoma (GBM) represents a highly invasive primary malignant tumor within the central nervous system (CNS). Temozolomide (TMZ), a first-line chemotherapy agent for GBM treatment, has significant limitations, including drug resistance, poor water solubility, a short half-life, and notable toxic side effects. The innovation of the TMZ dosage form is pivotal for enhancing its therapeutic efficacy. In this study, solid lipid nanoparticles (SLN) loaded with Angiopep-2 (A2) and TMZ (TMZ-A2SLN), a nanopolymer featuring a solid spherical morphology and a particle size of approximately 100 nm, were constructed. The combined effect of TMZ-A2SLN and small-interfering RNA (siRNA) that can knock down the expression of the *HOXB9* gene (siHOXB9) augmented the sensitivity of the glioma cell line U251 to TMZ. Under the combined effect, the viability of U251 cells was reduced by 77%. Meanwhile, the mortality rate increased by approximately 45%, and the cell apoptosis rate rose by around 36%. The number of cells arrested in the G2/M and S phases rose. Proteomic analysis indicates that TMZ-A2SLN might be implicated in the pro-inflammatory signaling cascade, tumor migration, invasion, and angiogenesis during the treatment of glioma cells. Moreover, *HOXB9* may play a crucial regulatory role in the PPAR signaling pathway, the neural signaling pathway, the phospholipase D signaling pathway, the IL-17 signaling pathway, mineral absorption, and other pathways during glioma cell treatment.

## 1. Introduction

Glioblastoma (GBM), a primary malignant tumor of the central nervous system (CNS), originates from glioblasts. Its annual incidence rate is approximately 3–8 cases per 100,000 individuals [1]. The management of GBMs demands a multidisciplinary strategy. This encompasses surgical resection, irradiation, systemic therapies, and supportive care [2,3]. Nevertheless, GBMs typically display a diffuse and infiltrative growth pattern. As a result, they are challenging to completely remove during surgery, highly prone to recurrence, and associated with a poor prognosis, thereby posing a well-recognized challenge in the field of neurosurgery [4]. Temozolomide (TMZ), as a first-line chemotherapy drug for the treatment of gliomas, only shows its anti-tumor activity after entering the human body. It is converted into the active compound 5-(3-methyltriazen-1-yl)-imidazole-4-carboxamide (MTIC), a TMZ active metabolite, through a non-enzymatic pathway. MTIC exerts its toxic effect by methylating the guanine in DNA, generating O-6-methylguanine (O6-MeG). Normally, guanine (G) pairs with cytosine (C), but O6-MeG may incorrectly pair with thymine (T) due to structural changes, and cells will activate the mismatch repair system (MMR) to correct this error. However, due to the structural abnormalities of O6-MeG, MMR can recognize the mismatch site but cannot effectively repair it, ultimately leading to DNA strand breakage [5,6,7]. However, these active metabolites have poor permeation through the blood–brain barrier. Ak Güliz used cetyl palmitate as the raw material and monocarboxylate transporter-1 and β-hydroxybutyric acid as the target molecules. Anti-cancer drugs carustine and TMZ are formulated into solid lipid nanoparticles (SLN) to enhance their anti-proliferative effects against GBM [8]. Jigar used the reverse-phase evaporation method to prepare TMZ-loaded PEGylated liposomes that provide optimal drug concentration at the tumor site [9]. In Xu’s work, poly(2-ethyl-2-oxazoline) conjugated to TMZ was synthesized and directly dissolved in PBS to form prodrug micelles, prolonging circulation time in vivo and increasing TMZ accumulation in glioblastoma [10]. Therefore, improving the therapeutic effect of TMZ on GBM has become a key issue currently faced by TMZ in the treatment of GBM, including enhancing the sensitivity of GBM to TMZ, increasing its solubility, prolonging its action time, increasing its targeting effect and the blood–brain barrier penetration ability, and reducing systemic toxicity.

In recent years, nanomaterials have received high attention and widespread application in the field of biomedical research [11]. With its extremely small size and special surface properties, polymer-assembled nanomedicine delivery systems have opened a new path in the field of GBM treatment. Researchers have chosen different types of nanomaterials and are committed to constructing a nanomedicine delivery system that can specifically recognize cancer cells, avoiding drug side effects and tolerance, and achieving controlled drug release [12]. Currently, extensively studied nanocarriers include inorganic nanoparticles, liposomes, and nanomagnetic particles [13], among others, and some have gradually advanced towards clinical applications. SLN can increase penetration across multiple biological barriers [14], making them widely used for brain administration. Wu constructed SLNs and nanostructured lipid carriers for co-delivery of vincristine and TMZ to develop the synergetic therapeutic action of the two drugs [15]. SLN can be administered through various routes in clinical practice, including oral administration, intravenous injection, and pulmonary administration. Based on the above advantages, SLN has broad application prospects in the research and development of drug carriers.

The *HOXB9* gene belongs to the homeobox (HOX) gene family and is the main control gene for development, playing a key role in regulating embryonic development, organ formation, and cell proliferation and differentiation [16]. The protein encoded by the HOX gene serves as a transcription factor and can form a complex positive and negative feedback loop regulatory network with upstream signaling molecules and downstream target genes [17,18,19]. It can also work together with other transcription factors to participate in organ or morphogenesis, cell adhesion and migration, and regulation of the cell cycle. When members of the HOX gene family are abnormally expressed at the wrong time or place, it can cause dysregulation of their regulatory functions and an imbalance in proliferation and differentiation, and may lead to the occurrence of tumors [20]. The mechanism of drug resistance in GBMs is exceptionally complex [21]. Although research on drug resistance genes and their products in malignant GBMs continues to be updated [22], the relationship between drug resistance genes and the treatment efficacy of GBMs in patients using TMZ is still unclear. Researchers have found that the *HOXB9* gene is highly expressed in patients with high-grade GBMs [23,24,25], and its expression level increases with the increase in pathological grade. At the same time, the *HOXB9* gene is associated with multiple drug-resistant molecules such as MGMT and P-gp. This indicates that the *HOXB9* gene has a significant impact on the occurrence, development, and chemotherapy resistance of GBMs, but its mechanism is still unclear.

This study developed a new formulation of TMZ-A2SLN based on SLN loaded with Angiopep-2 (A2) and found that the combination therapy of siHOXB9 with TMZ-A2SLN improved delivery while increasing glioma cell sensitivity to TMZ. Proteomics technology was applied to preliminarily explore the combined effect of the anti-tumor mechanism of the nanopolymer TMZ-A2SLN and siHOXB9. It provides a theoretical basis and technical support for the clinical treatment of GBMs.

## 2. Results

### 2.1. Preparation and Characterization of TMZ-A2SLN

**Figure 1:**
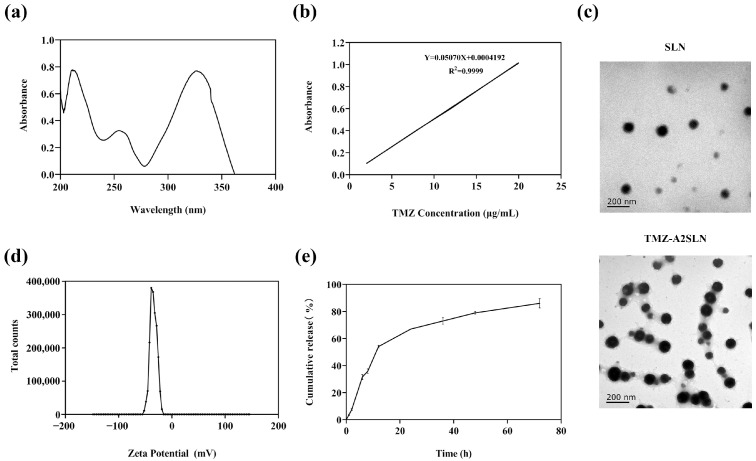
Preparation and characterization of TMZ-A2SLN. (**a**) The UV spectrogram of TMZ. (**b**) The standard curve of TMZ. (**c**) TEM image of TMZ-A2SLNs. (**d**) Zeta potential of TMZ-A2SLNs. (**e**) Release curve of TMZ-A2SLN in vitro (pH 6.8).

Establishing a TMZ content determination method is an important preliminary preparation for detecting the encapsulation efficiency and drug loading rate of nanocarrier systems. The results of the ultraviolet absorption peak scanning (Figure 1a) showed that the aqueous solution of temozolomide (TMZ) exhibited distinct absorption peaks at wavelengths of 327 nm, 254 nm, and 211 nm. Among these, the absorbance was relatively higher at 211 nm and 327 nm, indicating a stronger ultraviolet absorption response of TMZ at these two wavelengths. However, it should be noted that 211 nm is close to the ultraviolet end absorption region (typically 190–220 nm). This region is susceptible to interference from the ultraviolet absorption of the solvent itself and trace impurities in the system, and the instrumental detection stability is relatively poor, which may lead to a decrease in the precision and accuracy of the detection results. In contrast, 327 nm lies within the characteristic absorption range of TMZ, featuring high absorbance and being far from interference regions, thus offering superior detection stability and specificity. In line with the principle of “prioritizing the selection of characteristic wavelengths with strong absorption and low interference” in the quantitative detection by ultraviolet-visible spectrophotometry, 327 nm was determined as the detection wavelength in this study. Under measuring the absorbance of different concentrations of TMZ standard solutions, a plot is made with absorbance as the *y*-axis and drug concentration as the *x*-axis (Figure 1b), and the standard curve equation is as Y = 0.0507X + 0.0004, R^2^ = 0.9999 (Y: absorbance; X: concentration). The results indicate that TMZ has a good linear relationship with absorbance within the concentration range of 2–20 μg/mL.

After optimizing the synthesis of TMZ-A2SLN, the encapsulation efficiency and drug loading rate of TMZ in the nanoparticles were detected to be 74.27 ± 2.63% and 20.92 ± 5.44%, respectively. The TEM observation results showed that the constructed TMZ-A2SLN nanocomposite was a relatively uniform spherical structure with regular morphology and a diameter of around 100 nm (Figure 1c). Zeta potential was −36.7 mV (Figure 1d). The release efficiency of nanomedicines is an important indicator for verifying the sustained release performance of nanomedicines. Here, we used dynamic membrane dialysis to detect the release efficiency of TMZ-A2SLN in vitro. The release effect of TMZ-A2SLN in a pH = 6.8 buffer is shown in Figure 1e. TMZ-A2SLN was released slowly, with a drug release rate of 7.23% in the first 2 h, followed by continuous release. The total amount of drug released reached 84.81% at 72 h.

### 2.2. Evaluation of siRNA Transfection Effect

**Figure 2:**
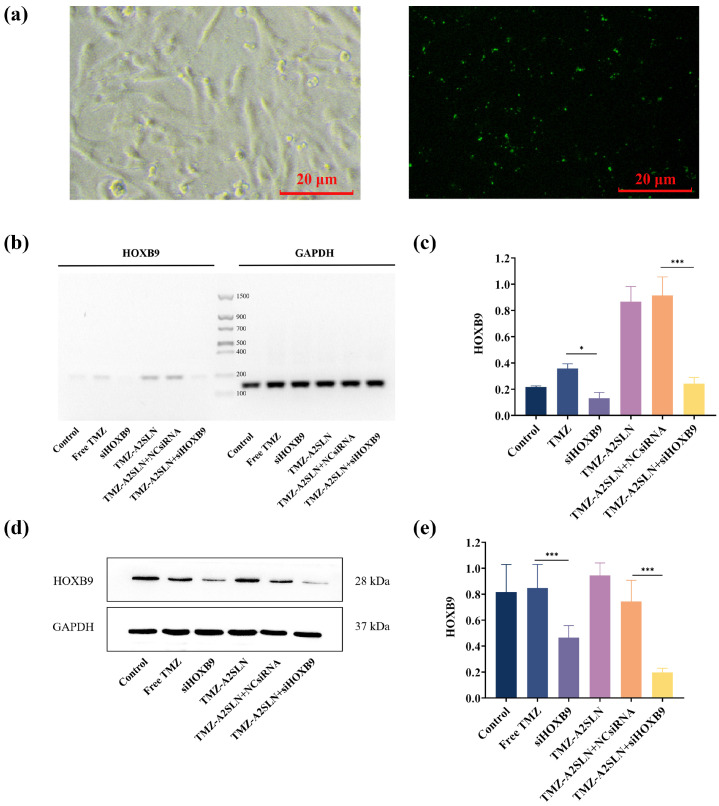
Effects of siRNA transfection. (**a**) Cell uptake of NC siRNA. (**b**) Effect of siRNA silencing on *HOXB9* mRNA levels. (**c**) Gray scale analysis of *HOXB9* mRNA levels. (**d**) Effect of siRNA silencing on *HOXB9* protein levels. (**e**) Gray scale analysis of *HOXB9* protein levels. * represents *p* < 0.05; *** represents *p* < 0.001.

To delineate the role of the *HOXB9* gene, we used specific siRNA to knock down its expression in U251 cells. In this study, a 24-well plate was used for transfection at a dose of 10 pmol/well siHOXB9. After transfection, the transfection efficiency of siHOXB9 was observed using a fluorescence microscope. The results are shown in Figure 2a, and bright green fluorescence can be observed under the fluorescence microscope, indicating that siHOXB9 has good transfection efficiency. RT-PCR and Western blot were used to detect the downregulation of *HOXB9* gene expression in U251 cells at mRNA and protein levels under different conditions (groups: PBS, Free TMZ, siHOXB9, TMZ-A2SLN, TMZ-A2SLN + NCsiRNA, TMZ-A2SLN + siHOXB9). As shown in Figure 2b,c, compared with the Free TMZ group, the siHOXB9 group showed a 25% decrease in *HOXB9* gene expression, indicating a significant difference. Compared with the TMZ-A2SLN + NCsiRNA group, the *HOXB9* gene expression was downregulated by 67% in the TMZ-A2SLN + siHOXB9 group, with a more significant difference. This indicates that compared to other groups, the TMZ-A2SLN + siHOXB9 group can significantly downregulate the expression of the target gene *HOXB9* (*p* < 0.001). Furthermore, Western blot was used to assess the ability of each group of compounds to downregulate *HOXB9* protein expression in U251 cells. As shown in Figure 2c,d, the TMZ-A2SLN + siHOXB9 group showed a 59% protein downregulation compared to the TMZ-A2SLN + NCsiRNA group, while the siHOXB9 group showed no significant difference compared to the Control group (*p* < 0.001). The above results demonstrate that adding the siRNA/RNA Trans Mate complex (siHOXB9) to a 24-well culture plate at a final siRNA concentration of 10 pmol/well and incubating at 37 °C for 24 h in a 5% CO_2_ incubator can successfully transfect and knock down the expression of the HOXB9 gene.

### 2.3. In Vitro Anti-Tumor Efficiency

**Figure 3:**
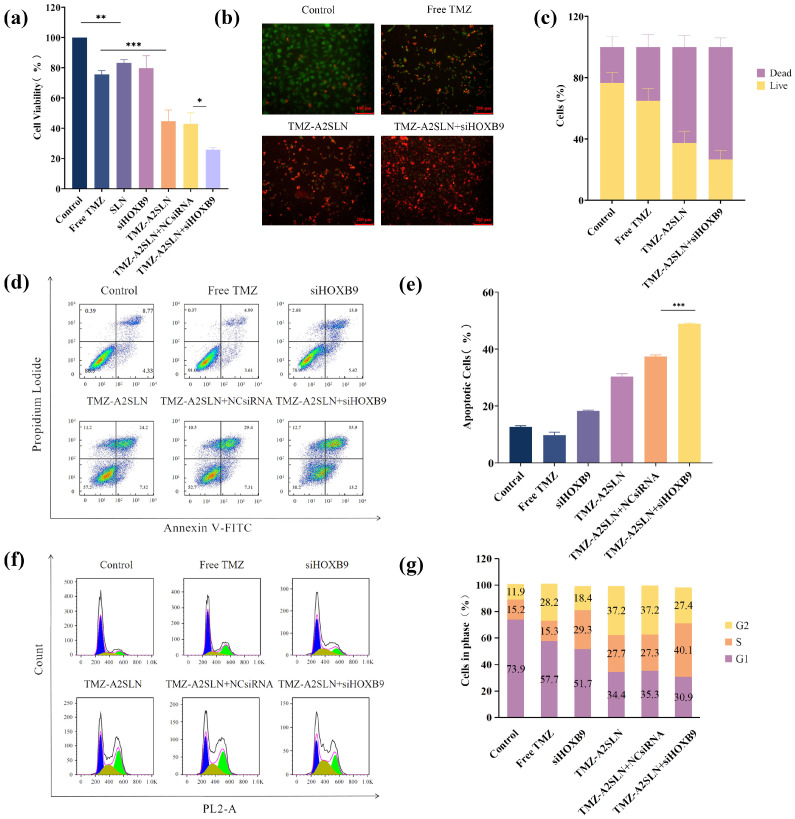
The combined effect of TMZ-A2SLN and siHOXB9 on U251 cells in vitro. (**a**) Effect of different treatments on the viability of U251 cells. (**b**) Effect of different treatments on cell death in U251 cells. (**c**) Analysis of the red fluorescence intensity. (**d**) Effect of different treatments on apoptosis in U251 cells. (**e**) Gray scale analysis of (**d**). (**f**) Effect of different treatments on the cell cycle of U251 cells. Blue: G1 phase, Yellow: S phase, Green: G2 phase. (**g**) Gray scale analysis of (**f**). * represents *p* < 0.05, ** represents *p* < 0.01, and *** represents *p* < 0.001.

Cell viability is an important indicator of cell survival status. The CCK-8 method was used to investigate the effect of TMZ-A2SLN combined with siHOXB9 on the activity of U251 cells with high expression of the *HOXB9* gene. As shown in Figure 3a, there was no significant difference between the SLN group and the control group in U251 cells, indicating that the nanocarrier SLN itself has low cytotoxicity to cells and has a relatively small impact on its viability. Compared with TMZ-A2SLN + NCsiRNA, there was no significant difference in U251 cell viability in the TMZ-A2SLN group. Therefore, the effect of siRNA transfection reagents on cell viability can be ignored. The viability of U251 cells in the TMZ-A2SLN group decreased by 31% compared to the free TMZ group, with a significant difference, indicating that TMZ encapsulated in the nanocarrier A2SLN has stronger cytotoxicity. It is speculated that A2 plays a targeted role in increasing the uptake rate of TMZ-A2SLN by cells. The cell activity of the TMZ-A2SLN + siHOXB9 group was 23%, which showed a significant inhibitory effect on tumor cell growth compared to other groups. The above results indicate that in U251 cells with high *HOXB9* gene expression, the combination of TMZ-A2SLN and siHOXB9 can effectively inhibit the viability of U251 cells.

Cell death is also an important indicator for evaluating the ability of nanomedicine delivery carriers. Live/Dead staining was performed on U251 cells treated with PBS, Free TMZ, TMZ-A2SLN, and TMZ-A2SLN + siHOXB9, and cell death was observed under a fluorescence microscope. The results showed (Figure 3b,c) that the TMZ-A2SLN + siHOXB9 group had the brightest red fluorescence, indicating more dead cells compared to other groups.

The apoptosis rate is also an important indicator for verifying the cytotoxic effect of nanomedicines on cells. We used the Annexin V-FITC/PI apoptosis detection method to investigate apoptosis of glioma cells under different conditions (groups: PBS, Free TMZ, siHOXB9, TMZ-A2SLN, TMZ-A2SLN + NCsiRNA, TMZ-A2SLN + siHOXB9). As shown in Figure 3d, the apoptosis rate of the control group (PBS) was 12.6%, while the apoptosis rate of the TMZ-A2SLN + siHOXB9 group was 48.9%. The apoptosis rates of the siHOXB9 group, Free TMZ group, TMZ-A2SLN, and TMZ-A2SLN + NCsiRNA group were 9.8%, 18.32%, 30.4%, and 37.4%, respectively. The apoptosis rate of the TMZ-A2SLN + siHOXB9 group increased by 36.3% compared to the control group (PBS) and 11.5% compared to the TMZ-A2SLN + NCsiRNA group, with significant differences. This indicates that the combined effect of TMZ-A2SLN and siHOXB9 can significantly promote apoptosis of glioma cells (*p* < 0.001). The apoptosis rate of the PBS control group was slightly higher than that of the Free TMZ group, but the data analysis results showed no significant difference.

The cell cycle includes G1, S, and G2/M phases, and the changes in the proportion of each phase can reflect the effect of drugs on cells. Through cell cycle experiments, it can be verified whether the combination of TMZ-A2SLN and siHOXB9 can increase the therapeutic effect of the chemotherapy drug TMZ. The detection results of flow cytometry (Figure 3e) showed that in U251 cells, the proportion of G2 phase in the free TMZ group increased by 16.3% compared to the control group. The proportion of the G2 phase in the TMZ-A2SLN group increased by 25.3% compared to the control group, indicating that using SLNs for TMZ delivery can block the cell cycle of U251 cells more in the G2 phase. Compared with the control group, the S phase of U251 cells in the siHOXB9 group increased by 14.1%, indicating that downregulation of *HOXB9* gene expression promoted U251 cell proliferation. Compared with the control group, the cell cycle of the TMZ-A2SLN + siHOXB9 group increased by 15.5% and 24.9% in G2 and S phases, respectively, with a total increase of 40.4%, which was significantly higher than that of other groups. The above results indicate that the combined action of TMZ-A2SLN and siHOXB9 can deliver TMZ into U251 cells and effectively downregulate the *HOXB9* gene expression, causing cell cycle arrest in the S and G2 phases and preventing cell transition to the division phase.

### 2.4. Proteomic Analysis and Identification of Differentially Expressed Proteins (DEPs)

The role of *HOXB9* in drug resistance in GBMs has been recognized by researchers [25]. Many researchers are using various methods to reduce the expression of *HOXB9* in tumor cells. However, the underlying mechanism of *HOXB9* in drug resistance has not yet been established. This study used IBT-based proteomic analysis to preliminarily explore the important regulatory role of *HOXB9* in synergy with TMZ-SLNs in U251 cells, and screened important signaling pathways related to *HOXB9* through GO, KEGG, and protein interaction networks.

**Figure A1:**
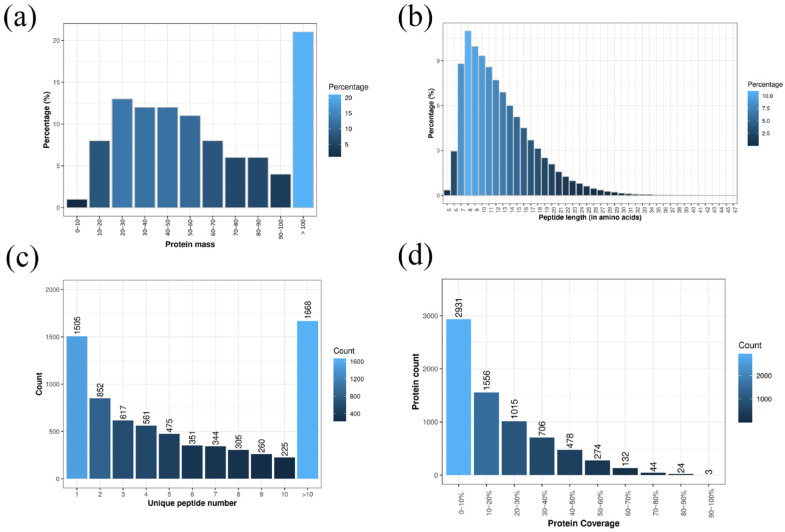
IBT data quality assessment information. (**a**) Range of protein molecular weight distribution. (**b**) Peptide length distribution. (**c**) Specific peptide number distribution map. (**d**) Protein coverage distribution.

Three groups were used, namely, PBS, TMZ-A2SLN, and TMZ-A2SLN + siHOXB9 groups. The quality evaluation of the identified peptide samples, labeling efficiency of protein n-terminus and lysine side chain groups, and analysis of CV repeat sequence distribution meet the subsequent analysis requirements as shown in Figure A1. A total of 753,739 secondary spectra were obtained using IBT quantitative proteomics technology. Under the 1% FDR filtering standard, a total of 58,911 peptide segments and 7163 proteins were identified.

**Figure 4:**
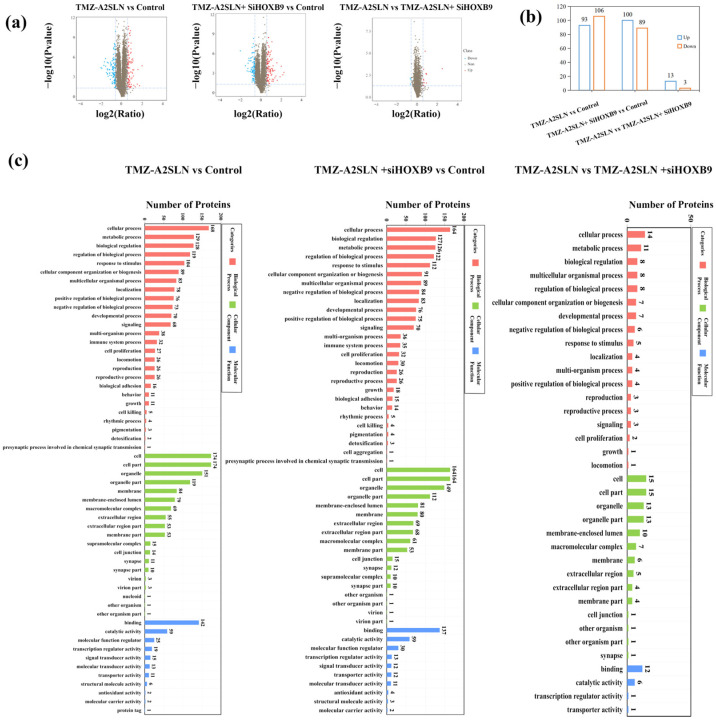
GO functional analysis of DEPs. (**a**) Volcano plot of DEPs. The *x*-axis and *y*-axis represent the log2-transformed fold change and the −log10-transformed significance, respectively. The *x*-axis and *y*-axis represent upregulated (red) and downregulated (blue) DEPs and DEP numbers, respectively. (**b**) Column chart of DEPs for the control group and treatment group. (**c**) GO functional classification of DEPs: TMZ-A2SLN vs. Control, TMZ-A2SLN + siHOXB9 vs. Control, TMZ-A2SLN vs. TMZ-A2SLN + siHOXB9.

In this protein analysis, the three main groups are: (1) TMZ-A2SLN vs. Control; (2) TMZ-A2SLN + siHOXB9 vs. Control; (3) TMZ-A2SLN vs. TMZ-A2SLN + siHOXB9. Under the screening criteria of FC > 1.5 and Q-value < 0.05, the differential protein results are shown in Figure 4a,b. Compared with the control group, TMZ-A2SLN treatment resulted in 93 proteins upregulated and 106 downregulated; after TMZ-A2SLN + siHOXB9 treatment, 100 proteins were upregulated and 89 downregulated. Compared with TMZ-A2SLN + siHOXB9, TMZ-A2SLN showed 13 proteins with higher expression levels and 3 with lower expression levels. Among these differentially expressed proteins (DEPs), numerous are involved in cell cycle regulation and apoptosis modulation. For instance, in the downregulated proteins of the TMZ-A2SLN vs. the Control group, we detected CCND3 (which regulates the G1→S phase transition), STIL (which modulates cell division progression), and POLD3 (which participates in DNA replication and damage repair and influences S phase progression). Meanwhile, we also identified anti-apoptotic proteins of the BCL-2 family, SQSTM1 (p62)—a cross-regulatory protein linking autophagy and apoptosis—and LGMN (involved in apoptotic body clearance, caspase activation, and the late execution phase of apoptosis). These data indicate that TMZ-A2SLN and TMZ-A2SLN + siHOXB9 exert their biological effects by regulating cell cycle- and apoptosis-related proteins.

Annotate and classify DEPs using GO and KEGG analysis. The GO enrichment of DEPs in the TMZ-A2SLN vs. Control (PBS) group is shown in Figure 4c. In molecular functional classification, binding and catalytic activities are enriched with more DEPs. In the cellular components, DEPs are enriched equally in both the Cell part and Cell, while Organelle, Organelle part, Membrane, and Membrane enclosed lumina are also enriched with more DEPs. Cellular Process, Metabolic Process, Biological Regulation, and Regulation of Biological Process are the largest subclasses in biological processes. The GO enrichment of DEPs between TMZ-A2SLN + siHOXB9 and the control group showed that molecular functional differential proteins were mainly enriched in binding, catalytic activity, and molecular functional regulation, indicating that TMZ-A2SLN + siHOXB9 stimulation causes differential changes in U251 cells at the molecular level. In the cellular components, entries such as cells, cell regions, organs and membranes, and membrane sealing cavities are enriched with more differential proteins. In biological processes, TMZ-A2SLN + siHOXB9 stimulation mainly affects protein changes in cellular processes, biological regulation, and metabolic processes. Screening of DEPs from the TMZ-A2SLN group and the TMZ-A2SLN + siHOXB9 group was performed, and the same and significantly different DEPs were selected for GO functional analysis. The results are shown in Figure 4c, and 16 differentially expressed proteins were identified. In terms of molecular function, the protein with the highest enrichment in binding function indicates that *HOXB9* may be involved in protein binding in U251 cells. In cellular components, more proteins were enriched in cells, cell regions, membranes, and membrane-enclosed cavities, indicating that *HOXB9* can increase GBM sensitivity to TMZ by regulating cell integrity. In biological processes, cellular processes, metabolic processes, biological regulation, and growth and development are the main GO functions; the enrichment of DEPs in these entries indicates the regulatory direction of *HOXB9* on GBMs.

**Figure 5:**
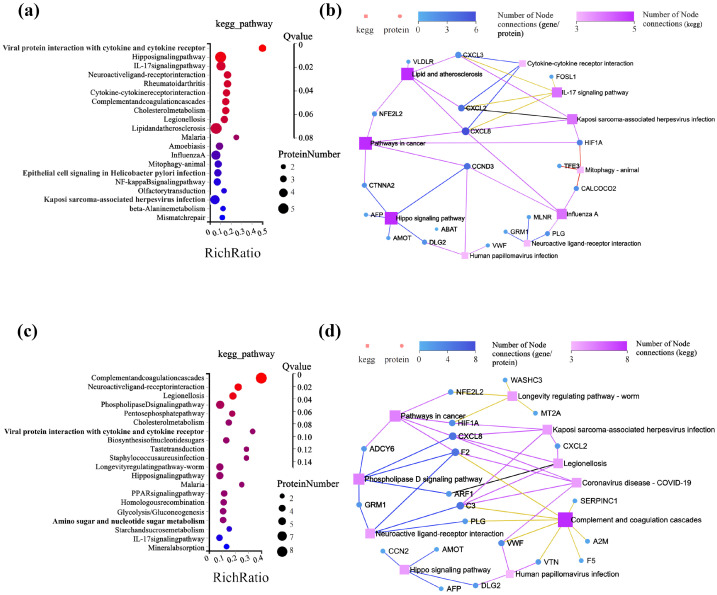
KEGG pathway analysis of DEPs. (**a**) Enriched bubble Figure of TMZ-A2SLN vs. Control. (**b**) Network diagram of TMZ-A2SLN + siHOXB9 vs. Control. (**c**) Enriched bubble Figure of TMZ-A2SLN + siHOXB9 vs. Control. (**d**) Network diagram of TMZ-A2SLN + siHOXB9 vs. Control. The color in the enrichment bubble map represents the enrichment significance value; as the color becomes redder, the significance value decreases.

The KEGG metabolic pathway showed that, in the TMZ-A2SLN vs. Control group (Figure 5a), DEPs were mainly enriched in pathways related to inflammation, such as the IL-17 signaling pathway, rheumatoid arthritis, NF-kappa B signaling pathway, and the complement and coagulation cascades. In addition, the Hippo signaling pathway, related to cancer inhibition, and Beta Alanine metabolism, related to cancer invasion, are also enriched. The KEGG network diagram (Figure 5b) showed that the Hippo signaling pathway, lipid and atherosclerosis, and cancer signaling pathways were the most enriched pathways. Proteins such as CXCL8 and CCND3 are associated with various pathways. Compared to the TMZ-A2SLN vs. Control group, the TMZ-A2SLN + siHOXB9 vs. Control group had more enriched DEPs in the phospholipase D signaling pathway, pentose phosphate pathway, cholesterol metabolism, and biosynthesis of nucleotide sugars, which affect tumor cell proliferation, invasion, and apoptosis (Figure 5c). In addition, it is also related to the PPAR signaling pathway associated with tumor resistance. The KEGG network diagram (Figure 5d) showed that the most enriched pathways are Complex and Coagulation, with a total of 8, followed by phospholipase D signaling and cancer signaling. Proteins such as CXCL8, C3, and F2 are enriched in multiple pathways.

## 3. Discussion

In this study, we aimed to investigate the effects of TMZ-A2SLN combined with siHOXB9 on U251 glioma cells. TMZ-A2SLN was prepared using a one-step method, which is a rapid and effortless approach. Encapsulating drugs in lipid nuclei reduces the fluidity of the encapsulated drugs, which is beneficial for controlling drug release [26]. The use of non-toxic surfactants such as poloxamer and phospholipids can increase their stability [27,28]. Polypeptide Angiopep-2 (A2) is one of the most used substances for nanocarrier modification, which can quickly bind to the A2 receptors expressed on the surface of GBMs through the blood–brain barrier, achieving the goal of targeted treatment of GBMs [29,30,31]. Therefore, A2 was connected to the synthesized TMZ-SLNs to enhance their targeting performance.

In the preliminary research, we extensively characterized TMZ-A2SLN, including studying its in vitro functional properties. When establishing the TMZ content determination method, 327 nm was selected as the detection wavelength. This detection result is within the maximum absorption wavelength range of TMZ in the literature (327~330 nm) [32]. On this basis, we measured the encapsulation efficiency of the nanocomposite at 74.27 ± 2.63% and the drug loading rate at 20.92 ± 5.44%, which are at the forefront of many studies [9,10,33]. The obtained nanocomposite has a relatively uniform spherical structure and regular morphology. Many studies have shown that TMZ raw materials are released rapidly in vitro, with about 80% released within 1 h and a maximum of 85.2% [34]. Here, TMZ-A2SLN has a significantly slow and controlled release effect, which can improve the bioavailability of TMZ drugs.

TMZ treats GBM by inducing DNA damage and cell apoptosis, but GBM’s resistance to TMZ is driven by multiple molecular mechanisms, posing significant challenges to treatment. The *HOXB9* gene shows elevated expression in patients with high-grade glioblastoma (GBM) and is associated with multiple drug-resistant mechanisms, including MGMT and P-gp. This study partially silenced *HOXB9* at the gene level in U251 cells using siHOXB9, followed by TMZ-A2SLN addition. With respect to cell activity and apoptosis, it was demonstrated that, compared with TMZ or TMZ-A2SLN alone, the combination of the two drugs significantly reduces U251 cell activity.

The cell cycle assay results showed that free temozolomide (TMZ) tends to induce G2 phase arrest because it is an alkylating agent that causes DNA damage. Cells initiate a stringent DNA damage checkpoint during the G2 phase, arresting cell cycle progression at G2 to facilitate DNA repair. If repair fails, apoptosis may occur. This is consistent with literature studies that TMZ can effectively block the cell cycle in the G2/M phase [35]. In contrast, siHOXB9 preferentially induces S phase arrest. *HOXB9* is a homeobox transcription factor that directly regulates genes involved in DNA replication (e.g., CDC6, MCM complex) and deoxynucleotide (dNTP) synthesis—processes critical for the S phase (DNA synthesis phase). Silencing *HOXB9* with siHOXB9 leads to stalled DNA replication forks and disrupted replication processes, triggering the S phase checkpoint and resulting in S phase arrest. Consequently, siHOXB9 exhibits a stronger S phase arrest effect than TMZ. The TMZ-A2SLN and TMZ-A2SLN + NCSIRNA groups showed the most potent G2 phase arrest. As a nanodelivery vector, TMZ-A2SLN is not merely a “transport tool”; it enhances the intracellular delivery efficiency of TMZ (avoiding drug efflux and increasing cytosolic concentration) and may improve drug accumulation in highly proliferative cells through targeting capabilities (e.g., binding to specific receptors on the surface of tumor cells). This amplifies TMZ-induced DNA damage, thereby strengthening G2 phase arrest compared to free TMZ. The TMZ-A2SLN + siHOXB9 group exhibited the strongest S phase arrest, which represents a key manifestation of the combined effects of the components. siHOXB9 directly impairs S phase DNA replication machinery, establishing a baseline S phase arrest. In cells arrested at the S phase, replication forks are inherently unstable and prone to collapse. Under such conditions, TMZ-induced alkylating damage further exacerbates replication stress, leading to complete replication fork collapse and the inability to proceed with DNA synthesis.

Additionally, this study employed proteomic analysis methods to preliminarily investigate the combined regulatory effects of *HOXB9* and TMZ-A2SLNs in U251 cells. Through GO analysis, KEGG pathway analysis, and protein–protein interaction network screening, key signaling pathways related to *HOXB9* and TMZ-A2SLN were identified, such as the PPAR signaling pathway associated with tumor drug resistance, the NF-κB signaling pathway linked to inflammation, and the Hippo signaling pathway involved in cancer suppression. This study lacks animal experiments, as the U251 cell tumor model is not ideal and is easily absorbed by mice/nude mice. The research team will continue to explore in depth from this study to provide more theoretical foundations and technical support for the clinical treatment of GBMs.

## 4. Materials and Methods

### 4.1. Materials and Reagents

U251 cells were gifted by Lingling Hou from Beijing Jiaotong University. The *HOXB9* primers, GAPDH primers, *HOXB9* siRNA, rabbit anti-*HOXB9* primary antibody, PBS, DMEM medium, fetal bovine serum, and RNA transfection reagents were all purchased from Sangon Biotech (Shanghai, China). Rabbit anti-GAPDH polyclonal antibody was purchased from Yeasen Biotechnology (Shanghai) Co., Ltd. (Shanghai, China). IRDye^®^ 680RD Goat anti-Rabbit was purchased from LI-COR (Lincoln, NE, USA). Angiopep-2 (A2) was synthesized from SynthBio (Hefei, Anhui, China). TMZ was purchased from Coolaber (Beijing, China). Dichloromethane was purchased from Aladdin (Shanghai, China). Soybean lecithin was purchased from Beijing Psaitong Biotechnology Co., Ltd (Beijing, China). Palmitic acid and poloxamer 188 were purchased from Shanghai Macklin Biochemical Technology Co., Ltd. (Shanghai, China). Chloroform, hydrochloric acid, and isopropanol were purchased from TGREAG (Beijing, China). The DMSO, TAE, protease inhibitor, and BCA protein concentration determination kit were purchased from LABLEAD (Beijing, China). The TBST, DEPC water, cell proliferation, and toxicity detection kit were purchased from Beijing Solarbio Science & Technology Co., Ltd. (Beijing, China). The cell cycle detection kit and apoptosis detection kit were purchased from UElandy (Suzhou, China). RIPA lysis solution and phosphatase inhibitor mixture were purchased from Beyotime (Shanghai, China). 2 × EasyTaq PCR SuperMix (+dye), Blue plus II Protein Marker, and Gelstain were all purchased from TransGen Biotech (Beijing, China). FastKing cDNA First Strand Synthesis Kit was purchased from TIANGEN BIOTECH (Beijing, China). PVDF film was purchased from Millipore (Bedford, MA USA).

### 4.2. TMZ Content Determination

Prepare TMZ solution (5 μg/mL), use ultrapure water as the blank control, and scan within the wavelength range of 200–400 nm using a UV-visible spectrophotometer (UV-2800A, Unico, Shanghai, China) to obtain the optimal detection wavelength for TMZ. Prepare standard solutions with concentrations of 2, 4, 8, 12, 16, and 20 μg/mL, then establish a standard curve for absorbance at the optimal detection wavelength.

### 4.3. Preparation of TMZ-A2SLN

TMZ-A2SLN was prepared via a one-step method. The specific procedures are as follows: Dissolve 50 mg of soybean lecithin and 500 mg of palmitic acid in 2 mL of dichloromethane; fully dissolve 250 mg of TMZ in 5 mL of DMSO, and mix the above two solutions to obtain the organic phase. Dissolve 200 μL of Poloxamer 188 emulsifier in 25 mL of ultrapure water to prepare the aqueous phase. Slowly add the prepared organic phase dropwise into the aqueous phase, with rapid stirring (1200 rpm) using a magnetic stirrer (GL-3250C, QILINBEIER, Nantong, Jiangsu, China) during the dropwise addition. The resulting solution was processed with a rotary evaporator (RE-52A, Shanghai Yarong, Shanghai, China). After the organic solvents were volatilized, a 1 μg/mL Angiopep-2 aqueous solution was added dropwise. Subsequently, the mixture was sonicated (XO, Nanjing Xian’ou Instrument Manufacturing Co., Ltd., Nanjing, Jiangsu, China) in an ice-water bath for 6 min, cooled until completely solidified to precipitate SLNs, and then filtered through a 2300-mesh sieve to obtain homogeneous TMZ-A2SLN.

### 4.4. Zeta Potentiometer Detection of TMZ-A2SLN Potential

The zeta potential of TMZ-A2SLN was measured using a Malvern Zetasizer Nano ZS90 (Malvern Instruments Limited, London, UK). With water as the medium, the temperature was set to 15 °C, followed by equilibration for 2 min.

### 4.5. Observation of TMZ-A2SLN Morphology Using Transmission Electron Microscopy

The morphology of the sample was observed using a transmission electron microscope (TEM) (Model: JEM-1400, JEOL, Akishima, Tokyo, Japan). One drop of the diluted sample was placed onto a 300-mesh copper grid. Subsequently, the sample was stained with a 2% (*w*/*v*) uranyl acetate solution before observation.

### 4.6. Encapsulation Rate and Drug Loading Rate

A UV-visible spectrophotometer was used for detection at the 327 nm wavelength. The amount of free TMZ (Wf) in the supernatant of TMZ-A2SLN, as well as the total TMZ content (Wt) in an equal volume of TMZ-A2SLN after demulsification with DMSO, was calculated based on the standard curve. The encapsulation efficiency (EE%) of TMZ in the nanoparticles was computed using the corresponding formula. An appropriate amount of freeze-dried TMZ-A2SLN (Freeze dryer: SPD2010-230, Thermo, Waltham, MA, USA) was accurately weighed, and the mass of the nanoparticles was recorded as Wn. Subsequently, a UV-visible spectrophotometer was employed to detect at 327 nm, and the mass of encapsulated TMZ (Wd) in the same mass of TMZ-A2SLN after demulsification with DMSO was calculated according to the standard curve. The drug loading (DL%) of TMZ in different nanoparticles was determined using the specified formula. Each sample was measured in triplicate.

The calculation formulas for encapsulation efficiency and drug loading capacity are as follows:

$$EE(\%)=\frac{Wt-Wf}{Wt}\times 100\%$$

$$DL(\%)=\frac{Wd}{Wn}\times 100\%$$

Wt is the concentration of TMZ in the suspension, Wf is the concentration of free TMZ, Wd is the mass of TMZ encapsulated in the nanoparticles, and Wn is the mass of TMZ-A2SLN.

### 4.7. In Vitro Release Rate

The in vitro drug release behavior of TMZ-A2SLN was investigated using the dynamic membrane dialysis method. TMZ-A2SLN solution containing 10.0 mg of TMZ was accurately measured and loaded into a dialysis bag (molecular weight cutoff: 12,000~14,000 Da). The dialysis bag was then placed into 100 mL of release medium (pH 6.8 phosphate-buffered saline, PBS) and subjected to oscillatory release at 37 °C and 100 rpm. At the predetermined time points of 1, 2, 4, 6, 8, 12, 24, 36, 48, and 72 h, 2 mL of the release medium was withdrawn, and an equal volume of pre-warmed release medium was supplemented simultaneously to maintain a constant volume. The concentration of TMZ in the collected samples was detected by a UV-visible spectrophotometer. A release curve was plotted with time as the abscissa and the cumulative release rate of TMZ as the ordinate. Each group was set with three parallel replicates.

### 4.8. Cell Line

U251 cells were cultured in DMEM medium containing 10% FBS and 1% penicillin–streptomycin solution. All cells were maintained at 37 °C with 5% CO_2_ in a cell incubator.

### 4.9. RNA Preparation and cDNA Synthesis

The procedure is as follows: U251 cells were lysed with TRIzol in the ultraclean stage and transferred to RNase-free EP tubes; vortexed for 30 s, then let stand on ice for 5 min. Add chloroform (Trizol/Chloroform = 5:1), vigorously vortex for 30 s, and let it stand on ice for 5 min to completely dissociate the nuclear protein complex. Centrifuge at 4 °C and 10,000× *g* for 15 min. Transfer the upper aqueous phase to the new EP tube. Add isopropanol (TRIZOL/Isopropanol = 1:1), invert and mix well about three times, let it stand on ice for 10 min, centrifuge at 4 °C and 10,000× *g* for 10 min, and a white gel-like precipitate can be seen. Discard the supernatant, add 1 mL of 75% ethanol (prepared with DEPC water), vigorously vortex for 30 s, and centrifuge at 4 °C for 5 min at 10,000× *g*. Remove the supernatant and air dry on an ultra-clean bench for 10–20 min. At this point, the white precipitate becomes transparent. Add 30–50 μL, blow, and mix well with RNase-free water. Concentrations were measured using a UV-visible spectrophotometer. cDNA synthesis, with a TIANGEN BIOTECH FastKing RT kit (with gDNase), was performed using 500 ng of RNA template according to the manufacturer’s recommendations.

### 4.10. Primer Design and Real-Time PCR (RT-PCR)

Gene expression changes were determined using RT-PCR. All primers were designed by Sangon Biotech. The final volume for each RT-PCR reaction was 20 µL, consisting of: 1 µL cDNA, 1 µL of each primer (100 µM), and 10 µL Premix (TransGen Biotech). Volumes were adjusted to 20 µL with RNase-free water. RT-PCR was performed with the Bio-Rad Laboratories RT-PCR Connect (RRID: SCR_008426, Heracles, CA, USA). using the following settings: an initial denaturation for 60 s at 94 °C, followed by 30 cycles of 30 s at 94 °C, 30 s at 58 °C (optimal temperature depending on the primer), and 30 s at 72 °C.

### 4.11. Gel Electrophoresis

Agarose (1.4 g) was added to 70 mL 1 × TAE buffer. The mixture was heated in a microwave oven for 2 min. Subsequently, the non-toxic nucleic acid stain GelStain (TransGen Biotech, Beijing, China) was added at a working concentration of 1×. After cooling to approximately 60 °C, the solution was poured into a gel plate with a gel thickness of 0.5 cm, and a comb was inserted to form gel holes. After the agarose gel was denatured, 10 μL of the samples was added to the gel holes and separated by electrophoresis. After electrophoresis was stopped, the gel was removed and imaged using a gel imaging analyzer (Gel Doc2000, BIO RAD, Heracles, CA, USA).

### 4.12. Western Blot

The procedure is as follows: Using lysis buffer (RIPA 1 mL, 100 × protease inhibitor 10 μL, 50 × phosphatase inhibitor 20 μL), lyse the cells and measure the protein concentration using the BCA method, ensuring a sample volume of 30 µg/15 µL per well. Add 5 × loading buffer to denature the protein, and adjust the system to 25 μL with water. Boil for 5 min, let it stand on ice, cool, stir well, and centrifuge for 5 min at 8000× *g*. The protein samples were subjected to SDS-PAGE electrophoresis using an electrophoresis instrument (164-5050, BIO RAD, Heracles, CA, USA), followed by transferring the protein onto a PVDF membrane using a membrane transfer instrument (165-8033, BIO RAD). Seal at room temperature for 1 h with TBST (0.05% Tween 20) containing 5% skim milk powder, incubate the primary antibody at 4 °C overnight with TBST (containing 5% skim milk powder) 1:2000 diluted, and incubate the secondary antibody at room temperature on a shaking table for 1 h with TBST (containing 5% skim milk powder) 1:5000 diluted. ECL method for development.

### 4.13. siRNA Transfection

Inoculate U251 cells at a density of 8 × 10^4^ cells per well in a 24-well plate and incubate at 37 °C in a 5% CO_2_ incubator for 24 h (cell confluence reaches 70% to 90%). Configure siRNA/RNA Trans Mate composites according to the manufacturer’s instructions. Add siRNA/RNA Trans Mate complexes to a culture plate containing cells and medium at a final concentration of 10 pmol/well of siRNA, with a positive control set for each dose. Incubate at 37 °C for 24 h in a 5% CO_2_ incubator. The next day, fluorescence was used to observe its transfection status. RT-PCR and Western blot were used to verify whether the *HOXB9* gene was knocked out.

### 4.14. Cell Proliferation

Inoculate U251 cells at a density of 3000 cells per well in a 96-well plate and incubate at 37 °C in a 5% CO_2_ incubator for 24 h. Group: PBS, Free TMZ, SLN, siHOXB9, TMZ-A2SLN, TMZ-A2SLN + NCsiRNA, and TMZ-A2SLN + siHOXB9. The dose of siHOXB9 and NCsiRNA is 20 pmol/well, and the TMZ dose is 250 μM. Add siRNA/RNA Trans Mate complex 6 h in advance, then add other drugs and incubate for 24 h. Add 10 μL CCK-8 solution to each hole. Incubate the culture plate in the incubator for 1.5 h and measure its absorbance at 450 nm using an enzyme-linked immunosorbent assay (Mμltiskan MK3, Thermo) reader. Vitality calculation is as follows:

$$Cell\;viability(\%)\;=\frac{A\;added-A\;blank}{A\;0\;added-A\;blank}\times 100\%$$

A (added): absorbance of pores with cells, CCK-8 solution, and drug solution. A (blank): absorbance of pores with culture medium and CCK-8 solution without cells. A (0 added): absorbance of pores with cells and CCK-8 solution, but no drug solution. Cell viability: cell proliferation or cytotoxicity.

### 4.15. Cell Live/Dead Staining

Inoculate U251 cells at a density of 1 × 10^5^ cells per well in a six-well plate and incubate at 37 °C in a 5% CO_2_ incubator for 24 h. Group: PBS, Free TMZ, TMZ-A2SLN, and TMZ-A2SLN/siHOXB9. Incubate according to 4.14. After incubation, clean twice with PBS. Mix the two tubes A (Live Green) and B (Dead Red) in the Live/Dead staining kit (UElandy, Suzhou, Jiangsu, China) by vortex mixing, and add 50 μL to each well staining solution, incubate at room temperature for 15 min. Observe cell staining under a fluorescence microscope.

### 4.16. Cell Cycle

Inoculate U251 cells at a density of 1 × 10^5^ cells per well in a six-well plate and incubate at 37 °C in a 5% CO_2_ incubator for 24 h. Group: PBS, Free TMZ, Free siHOXB9, TMZ-A2SLN, TMZ-A2SLN/NCsiRNA, and TMZ-A2SLN/siHOXB9. Incubate according to 4.14. After incubation, collect cells and wash once with pre-cooled PBS. Resuspend with pre-cooled 75% ethanol (prepared with anhydrous ethanol and ultrapure water) and fix overnight at −20 °C. Collect fixed cells and wash with pre-cooled PBS. Slowly resuspend the cells with propidium iodide staining solution and incubate at room temperature in the dark for 15–30 min. Use a flow cytometer to detect red fluorescence and light scattering in channels with excitation wavelengths of 535 nm and 615 nm. Using the Flowgo v10.8.1 software for cell DNA content analysis and light scattering analysis.

### 4.17. Cell Apoptosis

Prepare the preliminary cell samples according to method 4.16. Collect cells and follow the steps given in the apoptosis detection kit. Suspend the cells by adding 500 μL of binding buffer. After absorbing 5 μL of Annexin V-FITC and thoroughly mixing, add 5 μL of Propidium Iodide and mix again. React at room temperature and avoid light for 15 min, and then perform flow cytometry detection. Collect data and conduct data analysis.

### 4.18. Protein Analysis

According to the aforementioned method, stimulate U251 cells (divided into groups PBS, TMZ-A2SLN, TMZ-A2SLN + siHOXB9). Biological repetition three times. Extract protein according to the manufacturer’s (BGI Genomics Co., Ltd., Shenzhen, Guangdong, China) instructions, and then perform protein quantification using the Bradford method. Protein samples were digested with trypsin and then labeled with peptides using Isobaric Tags (IBT). (sample, label):control-1, 114; control-2, 115N; control-3, 115C; treated1-1, 117N; treated1-2, 117C; treated1-3, 118N; treated2-1, 118C; treated2-2, 119N; treated2-3, 119C.

Using a liquid phase system (LC-20AD, Shimadzu, Kyoto, Kyoto, Japan). Take the mixed 20 μg sample, perform liquid phase separation on the sample using a separation column (5 μm × 20 cm × 180 μm Gemini C18). The peptide segments that have undergone liquid phase separation are ionized by nano ESI sources and then transferred to a tandem mass spectrometer (Q-Exactive HF X, Thermo) for Data Dependent Acquisition mode detection. After converting the original mass spectrometry data into .mgf format files using corresponding tools, the protein identification software Mascot 2.3.02 was used to compare and search for identification in the UniProt protein database. FDR < 0.01. The statistical data of peptide quality can be found in the Figure A1. At least three identified proteins out of five replicates are considered for expression analysis.

### 4.19. Bioinformatics Analysis

The BLAST algorithm from the National Center for Biotechnology Information (NCBI) in the United States is used to obtain general information about the biological functions of identified proteins. Use Dr Tom 2.0 to annotate proteins using Gene Ontology (GO), and perform GO enrichment analysis using Fisher’s exact test. The Kyoto Encyclopedia of Genes and Genomes (KEGG) pathway analysis aims to investigate the high-level functionality and practicality of identified transcripts and proteins.

### 4.20. Statistical Analysis

The data generated in this study were all processed using the statistical software SPSS 21.0. Measurement data is expressed as mean ± standard deviation (Mean ± SD). Two-sample mean *t*-tests were used for comparing the means of two sets of analysis samples, and one-way ANOVA was used for comparing data between different groups. α = 0.05 is the inspection level. * indicates *p* < 0.05, ** indicates *p* < 0.01, *** indicates *p* < 0.001.

## 5. Conclusions

This study successfully prepared nanopolymer TMZ-A2SLN. The nanopolymer has a good morphology, presenting a solid spherical shape with a particle size of less than 200 nm, and has a good encapsulation effect on TMZ. The combined effect of TMZ-A2SLN together with siHOXB9 increased the sensitivity of U251glioma cells to TMZ. Under the combined effect, the viability of U251 cells significantly decreased, apoptosis increased, the mortality rate increased, and the number of cells in the cell cycle arrested in G2/M and S phases increased. TMZ may be involved in the pro-inflammatory signaling cascade, tumor migration, invasion, and angiogenesis during the treatment of GBMs. *HOXB9* may play an important regulatory role in the PPAR signaling pathway, neural signaling pathway, phospholipase D signaling pathway, IL-17 signaling pathway, mineral absorption, and other pathways during the treatment of GBMs.

## Data Availability

The original contributions presented in this study are included in the article. Further inquiries can be directed to the corresponding author.

## Abbreviations

The following abbreviations are used in this manuscript:

GBM	Glioblastoma
CNS	Central nervous system
SLN	Solid lipid nanoparticles
A2	Angiopep-2
TMZ	Temozolomide
TMZ-A2SLN	Solid lipid nanoparticles loaded with Angiopep-2 and TMZ
siRNA	Small interfering RNA
siHOXB9	Small interfering RNA that can knock down the expression of the *HOXB9* gene
MTIC	5-(3-methyltriazen-1-yl)-imidazole-4-carboxamide
O6-MeG	O-6-methylguanine
G	Guanine
C	Cytosine
T	thymine
MMR	Mismatch repair system
HOX	Homeobox
GO	Homeobox
KEGG	Kyoto Encyclopedia of Genes and Genomes

## Appendix A

This experiment consisted of three groups: PBS, TMZ-A2SLN, and TMZ-A2SLN + siHOXB9. A total of 753,739 secondary spectra were generated using IBT quantitative proteomics technology. Under the “1% FDR” filtering standard, a total of 58,911 peptide segments and 7163 proteins were identified. Evaluate the quality of the obtained peptide segments, including protein molecular weight distribution range, peptide length distribution, specific peptide number distribution map, and protein coverage distribution. Figure A1a shows that the molecular weights of the identified proteins are mainly distributed between 20 and 70 kDa, with the highest number of proteins having molecular weights between 20 and 30 kDa. As shown in Figure A1b, the length of peptide segments is mainly distributed between 7 and 11 amino acids, with the highest proportion of peptide segments being 8 amino acids. Figure A1c shows the distribution of the number of specific peptide segments identified by a single protein, and the protein credibility increases with the increase in the number of specific peptide segments. Figure A1d shows that proteins with a coverage of over 10% account for 41% of the total protein.
